# Year One of COVID-19 Pandemic: Effect on Presentation of Patients With Glaucoma in a Multi-Tier Ophthalmology Network in India

**DOI:** 10.3389/fopht.2022.900988

**Published:** 2022-07-06

**Authors:** Anthony Vipin Das, Sirisha Senthil

**Affiliations:** ^1^ Department of EyeSmart EMR & AEye, L V Prasad Eye Institute, Hyderabad, India; ^2^ Indian Health Outcomes, Public Health and Economics Research Center, L V Prasad Eye Institute, Hyderabad, India; ^3^ VST Glaucoma Centre, L V Prasad Eye Institute, Hyderabad, India

**Keywords:** COVID-19 pandemic, India, big data, glaucoma disorders, EMR

## Abstract

**Objective:**

To describe the demographics and clinical profile of patients with glaucoma presenting during the novel coronavirus (COVID-19) lockdown and unlock phases in India.

**Methods:**

This retrospective hospital-based comparative study included patients presenting between March 25, 2017, and March 31, 2021. All patients who presented with glaucoma disorders were included as cases. The demographic and clinical data of these glaucoma patients were collected using an electronic medical record system.

**Results:**

Overall, 34,419 patients (mean 47 per day) diagnosed with glaucoma diseases presented to the network and were included for analysis. The mean age of the patients was 54.16 ± 18.74 years and most were male (n=21,140; 61.42%) from the urban region (n=12,871;37.4%). On categorizing based on the timeline of the COVID-19 pandemic, most of the patients presented pre-COVID-19 (n=29,122; 84.61%), followed by a minority (n=175; 0.51%) during the lockdown and the rest (n=5,122; 14.88%) during unlock phase. An increasing number of patients with secondary glaucoma (n=82; 46.86%) and presenting from the local intra-city (n=82; 46.86%) was seen during the lockdown. There was a 6.6-fold increase in neovascular glaucoma and a 2.7-fold increase in lens induced glaucoma during the lockdown phase ((p<0.001) for both). There was a significant increase in subjects in 4^th^ decade (p<0.03) and a decrease in subjects in 7^th^ decade (p<0.008) during the lockdown period.

**Conclusion:**

The presentation of patients with glaucoma disorders to the hospital is evolving due to the COVID-19 pandemic. The footfalls of patients during the unlock regained to two-thirds of the pre COVID-19 level. During the lockdown, the older patients were less, there was an increase in younger patients and those with secondary glaucoma, and the majority presenting from within the city.

## Introduction

The ongoing novel coronavirus (COVID-19) pandemic has changed the world as we know it affecting more than 431 million individuals (1). Governments around the world were forced to quickly strategize to prevent the rapid spread of COVID-19 cases by enforcing lockdowns to restrict movement of individuals. The Government of India brought into effect a nation-wide lockdown to prevent the spread of the COVID-19 virus in a population of 1.3 billion people (2). Studies have shown that there was a sharp decline in patients accessing care during the lockdown period in India and there were a minor proportion of patients who presented to the emergency due to glaucoma related disorders (3, 4). The unlock 1.0 guidelines in India that were released from June 2020 ensured unrestricted movement of persons and goods (5). Rathi et al. shared the experience of unlock 1.0 on eyecare services which showed the highest reduction of patient footfalls in urban centers and there was an almost equal uptake of services by gender (6). The All India Ophthalmology Society published the preferred practice guidelines for glaucoma diseases that outlined triaging patients into high risk, medium risk and low risk based on the complaints and nature of the disease, provided guidelines on intraocular pressure measurement techniques and laid down protocols related to gonioscopy evaluation, investigations, lasers and surgical procedures (7). Studies from India have helped to identify the vulnerable patients such as females, pediatric age group, elderly population and rural geography who have not been able to access eyecare services (8–13). In this new normal we need to understand the trends of patients with chronic diseases such as glaucoma that warrants close monitoring and follow-up care. The authors describe a comparative report of the impact on the presentation of patients with glaucoma disorders to a large multi-tier ophthalmology network in India during the year one of the ongoing COVID-19 pandemic.

## Materials and Methods

### Study Design, Period, Location and Approval

This retrospective hospital-based comparative study included all patients diagnosed with glaucoma disorders presenting between March 25, 2017, and March 31, 2021, to a multi-tier ophthalmology network located in India (14). A standard consent form for electronic data privacy was signed by the patient or the parents or guardians of the patient at the time of registration. None of the identifiable parameters of the patient information were used for analysis of the data. The study adhered to the Declaration of Helsinki and was approved by the Institutional Ethics Committee. The clinical data of each patient who underwent a comprehensive ophthalmic examination was entered into a browser-based electronic medical records system (eyeSmart EMR) using a standardized template by trained ophthalmic personnel and supervised by an ophthalmologist (15).

### Data Retrieval and Processing

A total of 34,419 patients of all ages diagnosed with glaucoma disorders presented to the network during the study period and were included in this study. Non-glaucoma patients who attended the eyecare services were excluded from the study. The data of these patients were retrieved from the electronic medical record database and segregated in a single excel sheet (Microsoft XL^®^). Data on patient demographics such as age, gender, location, clinical presentation and ocular diagnosis were used for analysis. The patients were divided into primary and secondary glaucoma and were sub-divided based on the age and mechanism of the glaucoma. The excel sheet with the required data was then used for analysis using the appropriate statistical software. Standardized definitions were used for occupation, socio-economic status and geographic distribution (16). The study duration was divided into three categories, Pre COVID-19 between 25^th^ March, 2017 to 24^th^ March 2020, Lockdown phase between March 25^th^, 2020 to 31^st^ May, 2020 and Unlock phase between 1^st^ June, 2020 to March 31^st^, 2021 (17). The geographic distance was classified in relation to the eye care center at presentation. The patients presenting from the same location of the eye center were classified as “Intra-City”, those from the same state of the eye center were classified as “Intra-State” and the rest of the patients were classified as “Inter-State”. The demographic distribution and clinical presentation of the patients in these three categories were used for comparative analysis.

### Statistical Analysis

Descriptive statistics using mean ± standard deviation and median with inter-quartile range (IQR) were used to elucidate the demographic and clinical data using Microsoft Excel 2019 (Microsoft Corporation, Redmond, USA). Chi square test (StataCorp. 2015. Stata Statistical Software: Release 14. College Station, TX: StataCorp LP) was used for univariate analysis to detect significant differences in the distribution of demographics and clinical presentation of glaucoma patients during the Lockdown phase and the pre-COVID phase.

## Results

Overall, 34,419 patients diagnosed with glaucoma disorders presented during the study period. The distribution of glaucoma disorders was primary glaucoma in 20,099 (58.4%) patients, secondary glaucoma in 10,269 (29.84%), secondary developmental glaucoma in 1,471 (4.27%) patients, primary developmental glaucoma in 133 (0.39%) patients and unclassified in 2,447 (7.11%) patients.

### Overall Trend of Glaucoma

**Figure 1:**
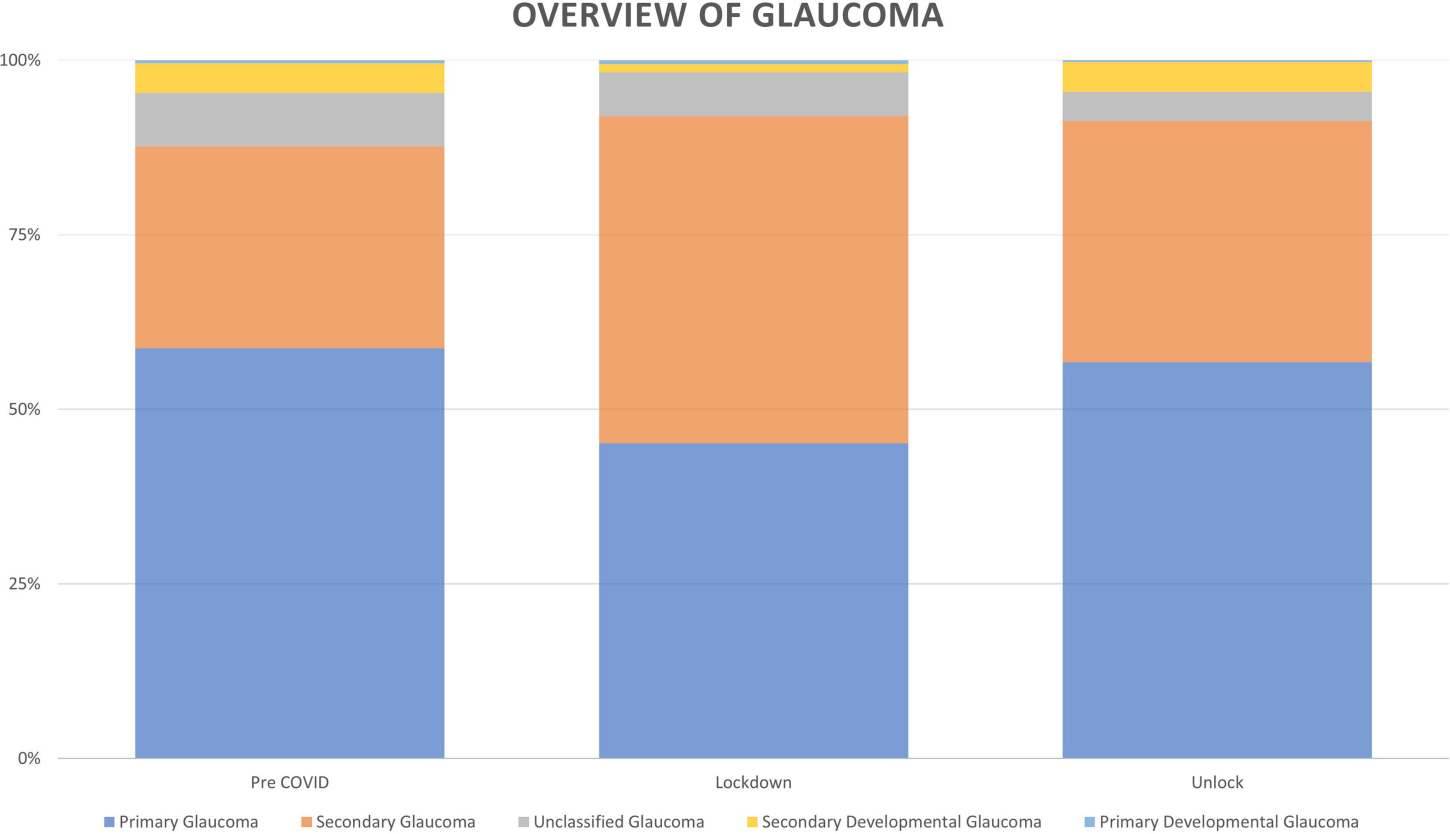
Distribution of patients with glaucoma disorders presenting during the Pre COVID-19, Lockdown and Unlock phase in India.

**Figure 2:**
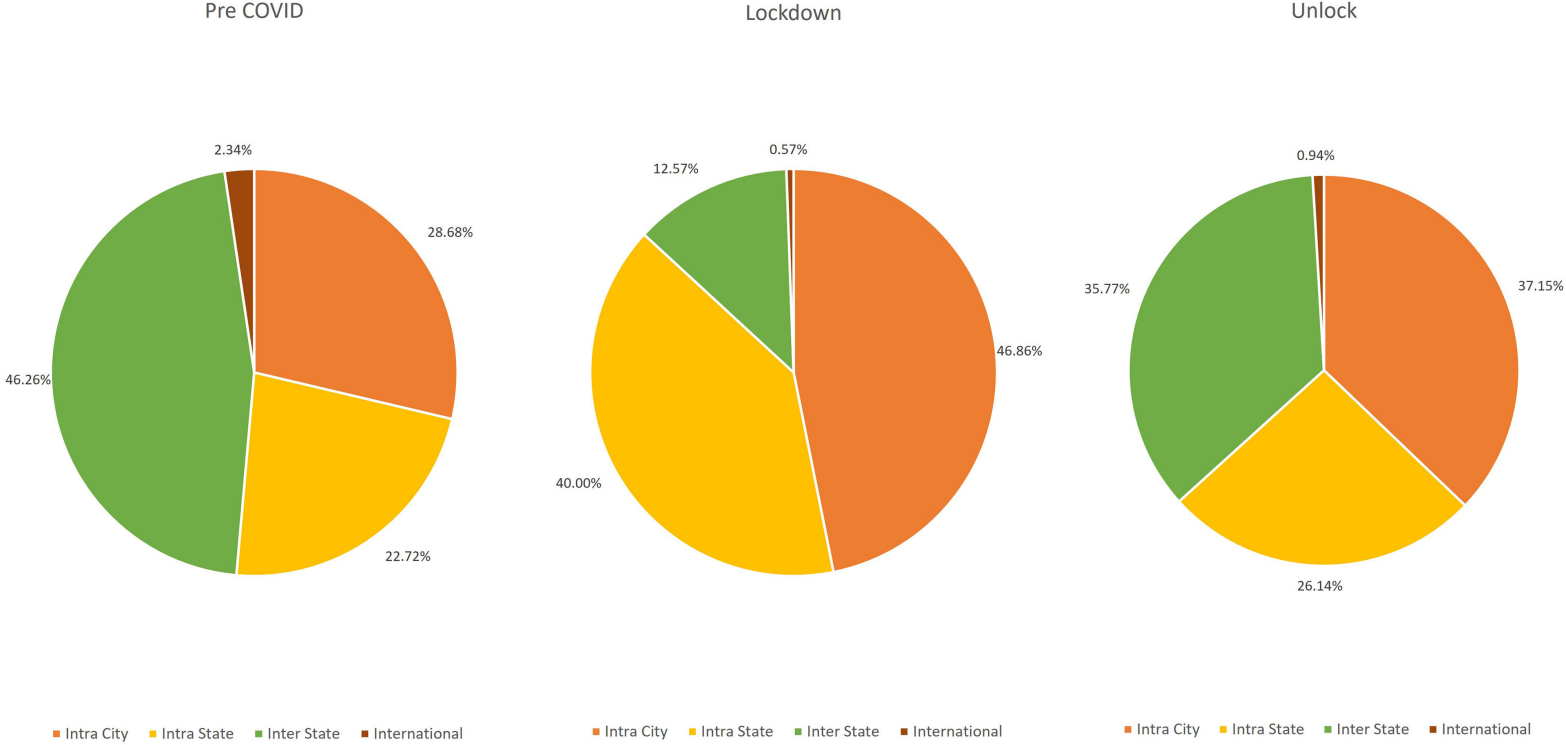
Geographic trends of patients with glaucoma disorders presenting during the Pre COVID-19, Lockdown and Unlock phase in India.

Overall, 34,419 patients diagnosed with glaucoma in one or both eyes presented during the study period. Compared to the pre COVID-19 phase with an average of 26.57 (29,122/1,096) patients per day, the number of patients seen during the lockdown phase with this diagnosis was significantly lower with an average of 2.57 (175/68) per day which increased to an average of 16.9 (5,122/303) during the unlock phase. There was no difference in the mean age of the patients (51.5 ± 20.16 years *vs* 51.92 ± 20.72 years) during COVID-19 phase (lockdown and unlock phases) as compared to the pre COVID-19 phase. The cohort of patients who presented were younger during the lockdown phase as compared to the pre COVID-19 phase in both primary (56.02 ± 16.89 years *vs* 53.03 ± 16.75 years) and secondary glaucoma (50.77 ± 21.71 years *vs* 46.09 ± 20.43 years). There was a decrease (8.8%) in the proportion of pediatric patients (≤16 years) during the COVID-19 phase as compared to the pre COVID phase (9.24%) which was not statistically significant (*p=0.34*). There was a small increase in access to care among the female (39.42% *vs* 38.43%; *p=0.36*) patients and a small decrease in male (60.58% *vs* 61.57%; *p=0.50*) patients, the difference was not statistically significant. There was an increase in access to care among the first-time visit (65.36% *vs* 59.27%; *p=0.000043*) patients and a decrease in follow up (34.64% *vs* 40.73%; *p=<0.00001*) patients during unlock phase which was statistically significant. There was an increase in patients with secondary glaucoma (34.94% *vs* 28.92%; *p=<0.00001*) patients and a decrease in patients with primary glaucoma (56.35% *vs* 58.75%; *p=0.09*) during the unlock phase. The trend of patients with glaucoma disorders over the three phases is detailed in **Figure 1**. With regards to place of origin, a proportional reduction of 27.18% (n=22/175 *vs* n=13,472/29,122) was seen in patients requiring inter-state travel and an increase was seen of 163.35% (n=82/175 *vs* n=8,352/29,122) in intra-city and 176.07% (n=70/175 *vs* n=6,616/29,122) in intra-state patients during the lockdown phase. There was a complete recovery in the proportion of outpatients to 115.07% (n=1,339/5,122 *vs* n=6,616/29,122) requiring intra-state travel and an incomplete recovery to 40.02% (n=48/5,122 *vs* n=682/29,122) for international patients during the unlock phases. The detailed comparison of the geographic presentation in all the three phases is described in **Figure 2**. A comparison of Pre COVID-19, Lockdown and Unlock phase of patients diagnosed with glaucoma disorders in India is detailed in **Table 1**.

**Table 1:** Demographic distribution of glaucoma patients during the Pre-, Lockdown and Unlock phases of the COVID-19 pandemic in India*.

Parameters	N	%	Pre-COVID	%	Lockdown	%	Unlock	%	*p value*
Total Patients	34419	100%	29122	84.61%	175	0.51%	5122	14.88%	***<0.00001***
Male	21140	61.42%	17931	61.57%	99	56.57%	3110	60.72%	0.50
Female	13279	38.58%	11191	38.43%	76	43.43%	2012	39.28%	0.37
0-10 yrs	2306	6.70%	1947	6.69%	12	6.86%	347	6.77%	0.93
11-20 yrs	1485	4.31%	1288	4.42%	6	3.43%	191	3.73%	0.53
21-30 yrs	1889	5.49%	1610	5.53%	11	6.29%	268	5.23%	0.68
31-40 yrs	2259	6.56%	1877	6.45%	17	9.71%	365	7.13%	0.1
41-50 yrs	4521	13.14%	3785	13.00%	34	19.43%	702	13.71%	***0.03***
51-60 yrs	7496	21.78%	6234	21.41%	40	22.86%	1222	23.86%	0.7
61-70 yrs	9151	26.59%	7807	26.81%	43	24.57%	1301	25.40%	0.6
71-80 yrs	4465	12.97%	3828	13.14%	10	5.71%	627	12.24%	***0.008***
81-90 yrs	806	2.34%	707	2.43%	2	1.14%	97	1.89%	0.27
91-100 yrs	41	0.12%	39	0.13%	0	0.00%	2	0.04%	NA
Adult	31261	90.82%	26430	90.76%	160	91.43%	4671	91.19%	0.94
Paediatric	3158	9.18%	2692	9.24%	15	8.57%	451	8.81%	0.77
Fresh	20724	60.21%	17262	59.27%	89	50.86%	3373	65.85%	0.24
Follow Up	13695	39.79%	11860	40.73%	86	49.14%	1749	34.15%	0.15
Paying	27225	79.10%	22839	78.43%	134	76.57%	4252	83.01%	0.83
Non-paying	7194	20.90%	6283	21.57%	41	23.43%	870	16.99%	0.63
Urban	12871	37.40%	11103	38.13%	52	29.71%	1716	33.50%	0.11
Rural	12142	35.28%	10180	34.96%	65	37.14%	1897	37.04%	0.67
Metropolitan	9406	27.33%	7839	26.92%	58	33.14%	1509	29.46%	0.17
Intra City	10337	30.03%	8352	28.68%	82	46.86%	1903	37.15%	***0.0002***
Intra State	8025	23.32%	6616	22.72%	70	40.00%	1339	26.14%	***0.000055***
Inter State	15326	44.53%	13472	46.26%	22	12.57%	1832	35.77%	***<0.00001***
International	731	2.12%	682	2.34%	1	0.57%	48	0.94%	0.13

### Specific Trends of Glaucoma

**Figure 3:**
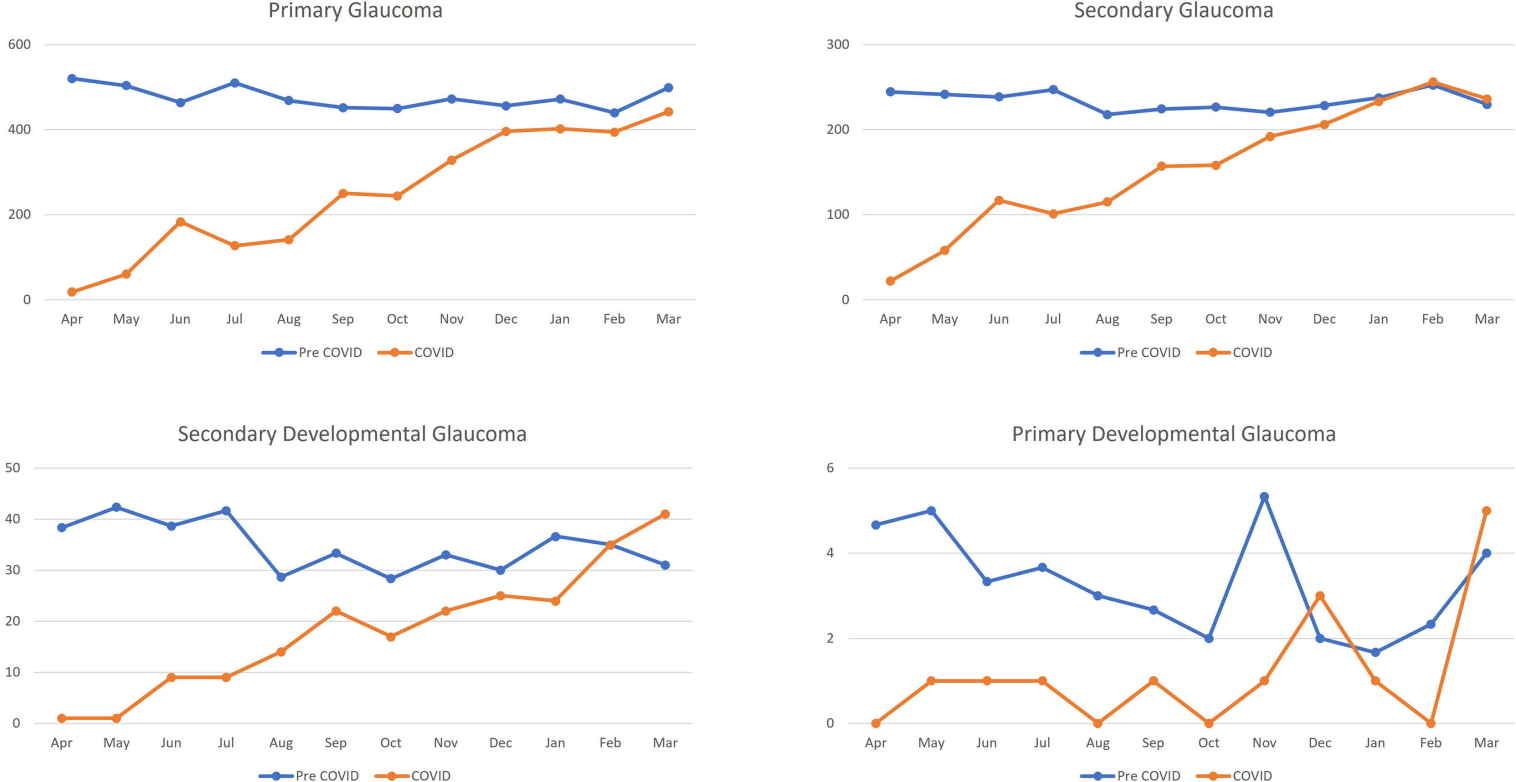
Monthly trends of patients with glaucoma disorders presenting during the Pre COVID-19, Lockdown and Unlock phase in India. *x-axis denotes the month of the year and y-axis denotes the patients per month.

The averaged-annual frequency of glaucoma patients during the pre-COVID-19 phase was 9,707 which reduced to 5,297 during the COVID-19 phase, while the averaged-monthly frequency also reduced from 809 to 441 patients. For primary glaucoma, the average-annual frequency decreased from 5,703 to 2,985 patients, while the averaged-monthly frequency decreased from 475 to 249 patients and showed a gradual increasing trend never exceeding the pre COVID-19 monthly average. In secondary glaucoma, the averaged-annual frequency decreased from 2807 to 1851 patients, and from 234 to 154 patients for the averaged-monthly frequency and showed a gradual increasing trend matching the pre COVID-19 monthly average by January 2021. For secondary developmental glaucoma, the averaged-annual frequency decreased from 417 to 220 patients, while the averaged-monthly frequency reduced from 35 to 18 patients and exceeded the pre COVID-19 monthly average by March 2021. The averaged-annual frequency of patients with primary developmental glaucoma decreased from 40 to 14 patients, whereas the averaged-monthly frequency reduced from 3 to 1 patient and showed a gradual increasing trend to exceed the pre COVID-19 monthly average by March 2021. The detailed comparison of the monthly trends of patients with glaucoma disorders in the pre COVID-19 and COVID-19 phase is described in **Figure 3**. A comparison of Pre COVID-19, Lockdown and Unlock phase of the glaucoma disorders is detailed in **Table 2**.

**Table 2:** Distribution of glaucoma subtypes during the Pre-, Lockdown and Unlock phases of the COVID-19 pandemic in India*.

Glaucoma Subtypes	N	%	Pre-COVID	%	Lockdown	%	Unlock	%
**Primary Glaucoma**	**20099**	**58.40%**	**17114**	**85.15%**	**79**	**0.39%**	**2906**	**14.46%**
Primary Open Angle Glaucoma	6420	31.94%	5576	32.58%	21	26.58%	823	28.32%
Primary Angle Closure Glaucoma	5238	26.06%	4397	25.69%	26	32.91%	815	28.05%
Primary Angle Closure	3742	18.62%	3148	18.39%	13	16.46%	581	19.99%
Primary Angle Closure Suspect	1237	6.15%	1015	5.93%	7	8.86%	215	7.40%
Juvenile Open Angle Glaucoma	932	4.64%	791	4.62%	3	3.80%	138	4.75%
Primary Congenital Glaucoma	921	4.58%	801	4.68%	3	3.80%	117	4.03%
Ocular Hypertension	893	4.44%	774	4.52%	3	3.80%	116	3.99%
Normal Tension Glaucoma	630	3.13%	541	3.16%	1	1.27%	88	3.03%
Acute Angle Closure	86	0.43%	71	0.41%	2	2.53%	13	0.45%
**Secondary Glaucoma**	**10269**	**29.84%**	**8418**	**81.97%**	**82**	**0.80%**	**1769**	**17.23%**
Pseudoexfoliation Glaucoma	2269	22.10%	1874	22.26%	3	3.66%	392	22.16%
Neovascular Glaucoma	1483	14.44%	1180	14.02%	19	23.17%	284	16.05%
Glaucoma in Pseudophakia	1165	11.34%	962	11.43%	6	7.32%	197	11.14%
S/p Vitreo-retinal Surgery Glaucoma	1093	10.64%	898	10.67%	4	4.88%	191	10.80%
Secondary Glaucoma (Unknown)	838	8.16%	658	7.82%	9	10.98%	171	9.67%
Steroid Induced Glaucoma	742	7.23%	621	7.38%	3	3.66%	118	6.67%
Traumatic Glaucoma	641	6.24%	543	6.45%	9	10.98%	89	5.03%
Lens Induced Glaucoma	566	5.51%	431	5.12%	19	23.17%	116	6.56%
Post Keratoplasty	545	5.31%	478	5.68%	3	3.66%	64	3.62%
Uveitic Glaucoma	353	3.44%	283	3.36%	3	3.66%	67	3.79%
ICE Syndrome	205	2.00%	175	2.08%	2	2.44%	28	1.58%
Glaucoma in aphakia	190	1.85%	158	1.88%	0	0.00%	32	1.81%
Pigmentary Glaucoma	102	0.99%	86	1.02%	1	1.22%	15	0.85%
Fuch's Heterochromic Iridocyclitis	50	0.49%	46	0.55%	0	0.00%	4	0.23%
Posner Scholssman Syndrome	27	0.26%	25	0.30%	1	1.22%	1	0.06%
**Unclassified**	**2447**	**7.11%**	**2220**	**90.72%**	**11**	**0.45%**	**216**	**8.83%**
Absolute Glaucoma	1231	50.31%	1168	52.61%	6	54.55%	57	26.39%
S/p Trabeculectomy	828	33.84%	710	31.98%	3	27.27%	115	53.24%
Total Glaucomatous Optic Atrophy	209	8.54%	198	8.92%	2	18.18%	9	4.17%
S/p Ahmed Glaucoma Valve	160	6.54%	129	5.81%	0	0.00%	31	14.35%
Plateau Iris Syndrome	19	0.78%	15	0.68%	0	0.00%	4	1.85%
**Secondary Developmental Glaucoma**	**1471**	**4.27%**	**1251**	**85.04%**	**2**	**0.14%**	**218**	**14.82%**
Microphthalmos	776	52.75%	675	53.96%	1	50.00%	100	45.87%
Axenfeld Rieger Syndrome	143	9.72%	120	9.59%	1	50.00%	22	10.09%
Aniridia	140	9.52%	118	9.43%	0	0.00%	22	10.09%
Secondary Developmental Glaucoma	132	8.97%	101	8.07%	0	0.00%	31	14.22%
Sturge Weber syndrome	131	8.91%	112	8.95%	0	0.00%	19	8.72%
Microspherophakia	101	6.87%	84	6.71%	0	0.00%	17	7.80%
Marfan's Syndrome	44	2.99%	41	3.28%	0	0.00%	3	1.38%
Nanophthalmos	4	0.27%	0	0.00%	0	0.00%	4	1.83%
**Primary Developmental Glaucoma**	**133**	**0.39%**	**119**	**89.47%**	**1**	**0.75%**	**13**	**9.77%**
Primary Developmental Glaucoma	133	100.00%	119	100.00%	1	100.00%	13	100.00%
**Grand Total**	**34419**	**100.00%**	**29122**	**84.61%**	**175**	**0.51%**	**5122**	**14.88%**

*added as supplementary file.

## Discussion

This study sought to describe the demographics and clinical profile of patients with glaucoma disorders presenting during the pre-COVID-19, lockdown phase and unlock phase in India. The findings of this study suggest that the mean footfalls of patients with glaucoma disorders showed a sharp decline during the lockdown phase and regained to two-thirds of the pre COVID-19 level during the unlock phase. There was an increasing trend seen in males, younger patients, those from higher socio-economic status and higher volumes from the local region. There was a decreasing trend seen among females, lower socio-economic status and presentation from outside the state. There was significant increase in presentation of new patients during the unlock phase and significant increase in presentation of secondary glaucoma both during the lockdown and unlock phases. As shown in **Table 2**, among the secondary glaucoma, neovascular glaucoma and lens induced glaucoma were significantly more.

Our experience in the early phase of the pandemic showed that the patients with glaucoma disorders were triaged as emergency (4.13%), urgent (19.59%) and routine (7.5%) patients. The major proportion of the emergency patients were due to phacomorphic glaucoma, raised IOP >40 mm Hg and acute angle closure (3). The current study shows an increase in the patients presenting with secondary glaucoma during the lockdown phase. There was an increase of 162.02% in the proportion of patients with secondary glaucoma during the lockdown phase and continued to be higher of 119.42% than the pre COVID-19 footfalls during the unlock phases. There was a decrease of 23.16% in the patients presenting with primary glaucoma during the lockdown phase which recovered to 96.57% of the pre COVID-19 footfalls during the unlock phase.

There was a decrease in the number of new patients during the lockdown phase and an increase in the follow up patients. The restrictions imposed due to the lockdown prevented people from travelling to the hospital which explains the decrease in the presentation of the new patients. There was also a 163.38% increase of patients presenting from intra city and a 176.07% increase of patients coming from within the same state during the lockdown phase. There was a 72.82% drop in the patients presenting from outside the state due to the travel restrictions that were in force in different parts of India. Muralikrishnan et al. described a decrease in the patients with glaucoma during the pandemic period (0.8% *vs* 1%) as compared to the pre-pandemic period but saw an increase in the lens induced glaucoma (0.03% *vs <*0.01%) during the pandemic period (18). Ayub et al. described a 92.52% decrease in their outpatient clinical visits among glaucoma patients in the first year of the pandemic (March 2020-February 2021) (19). It is interesting to note that the recovery of the patients with glaucoma during the unlock phase was two-thirds of the pre COVID-19 levels indicating a partial recovery of the access to eye care services.

The COVID-19 pandemic has forced a new normal and there have been major modifications implemented by glaucoma practices such as enhanced disinfection protocols enhancing safety of the patients and staff, reorganization of patient flow thereby triaging care for the needy and the use of technology to harness the benefit of tele-glaucoma systems (20). The use of teleconsultations has seen an exponential rise during the COVID-19 pandemic. There is significant benefit in the use of such a system to help monitor medication compliance, post-surgical follow-up and address new complaints if any from patients with glaucoma (21). As glaucoma is a chronic ocular disorder that needs close monitoring of the effect of medications on the intraocular pressure and the progression of visual fields, it is important to be accessible to the patient in critical times such as this evolving pandemic. Patients must be educated based on the current experience on the importance of the use of technology tools such as teleconsultation to continue to stay in touch with the eye care provider for efficient continuity of care. Vulnerable groups must be specifically addressed ahead of time with the waxing and waning of the waves of the pandemic with the onset of newer variants of the COVID-19 virus. Glaucoma is a chronic progressive disease that results in irreversible vision loss when untreated or unmonitored. Hence patients with such conditions need monitoring and continued treatment irrespective of the challenges posed by the pandemic.

In conclusion, the authors present their experience on the demographic and clinical presentation of patients with glaucoma disorders presenting to a multi-tier ophthalmology network in India during the COVID-19 pandemic. The vulnerable patient groups of females, lower socio-economic status and patients travelling from outside the state need focused attention in times of crisis such as this. The footfalls of patients during the unlock phase regained to two-thirds of the pre COVID-19 level. There was an increase in patients with secondary glaucoma and in those presenting from the local intra city during the lockdown phase. With diabetic and elderly patients noted to be at higher risk for COVID, these patients possibly did not access health care which possibly led to serious consequence of increase in neovascular glaucoma and lens induced glaucoma during the time of lockdown.

## Data Availability Statement

The raw data supporting the conclusions of this article will be made available by the authors, without undue reservation.

## Ethics Statement

The studies involving human participants were reviewed and approved by Institutional Review Board, LVPEI. Written informed consent to participate in this study was provided by the participants’ legal guardian/next of kin.

## Author Contributions

All authors listed have made a substantial, direct, and intellectual contribution to the work and approved it for publication.

## Funding

This study was funded by the Hyderabad Eye Research Foundation.

## Conflict of Interest

The authors declare that the research was conducted in the absence of any commercial or financial relationships that could be construed as a potential conflict of interest.

## Publisher’s Note

All claims expressed in this article are solely those of the authors and do not necessarily represent those of their affiliated organizations, or those of the publisher, the editors and the reviewers. Any product that may be evaluated in this article, or claim that may be made by its manufacturer, is not guaranteed or endorsed by the publisher.
